# Allelopathy and Identification of Allelochemicals in the Leaves of *Hakea decurrens* subsp. *physocarpa* W.R. Barker

**DOI:** 10.3390/plants14111646

**Published:** 2025-05-28

**Authors:** Laura Nogales, Juan Carlos Alías, José Blanco-Salas, Ismael Montero-Fernández, Natividad Chaves

**Affiliations:** Department of Plant Biology, Ecology and Earth Sciences, Faculty of Science, Universidad de Extremadura, 06080 Badajoz, Spain; lnogalesg@unex.es (L.N.); jalias@unex.es (J.C.A.); blanco_salas@unex.es (J.B.-S.); ismontero@unex.es (I.M.-F.)

**Keywords:** invasive plants, allelopathy, allelochemicals, *Hakea decurrens* subsp. *physocarpa*

## Abstract

*Hakea decurrens* subsp. *physocarpa* is an invasive species from Australia, with morphological, physiological and ecological features that help it colonize and settle outside of its natural habitats. One of these characteristics is allelopathy, which is an interaction that grants a clear competitive advantage to invasive species that has not been studied in *H. decurrens* subsp. *physocarpa*. With the aim of understanding the ecological relationships that take place in habitats invaded by this species, it is especially important to know the allelopathic potential of *H. decurrens* subsp. *physocarpa* and the compounds that would be involved in this interaction. To this end, the present study quantified the allelopathic activity of the aqueous extract of leaves gathered on four different occasions in the year, as well as of the compounds present in these extracts. The obtained results show a negative effect of *H. decurrens* subsp. *physocarpa* samples collected in March, June, September, and December on the germination and growth of *Lactuca sativa*. Although the negative effect was observed with all extracts, the extract of leaves gathered in September showing the greatest effect on germination (I_50_ = 0.08 g/mL), and that of leaves collected in June presented the greatest effect on root size (I_50_ = 0.05 g/mL). As for the composition of these extracts, nine compounds were identified and quantified through HPLC: arbutin, mesaconic acid, isotachioside, 1-O-vanilloyl-beta-D-glucose, syringic acid-4-beta-D-glucopyranoside, quercetin 3-robinobioside-7-glucoside, quercetin 3-rhamninoside, rutin, and isorhamnetin-3-O-rutinoside. There is a correlation between the quantified parameters and the quantity of these compounds in the extracts, but it is difficult to attribute the allelopathic activity of *H. decurrens* subsp. *physocarpa* to a particular compound, since this activity may depend on the combination of these compounds. In conclusion, this work demonstrates that the leaves of the invasive species *H. decurrens* subsp. *physocarpa* have allelopathic potential, and their toxicity could be due to the combined action of these compounds, which should be analyzed in future studies.

## 1. Introduction

**Figure 1:**
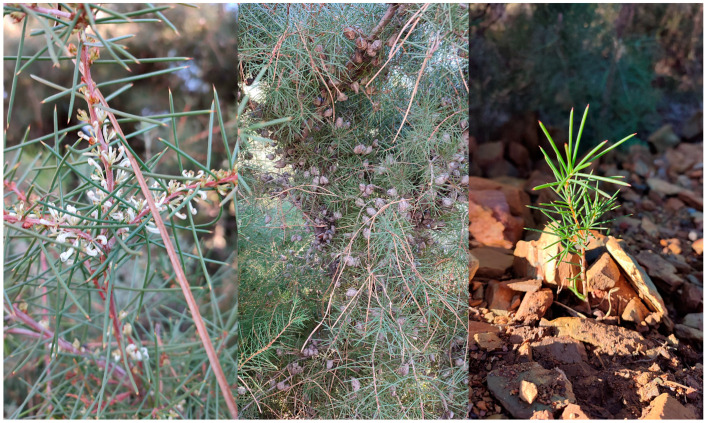
Details of the stem, leaves, flowers, fruits, and a sapling of *Hackea decurrens* subsp. *physocarpa*. Own source.

Exotic invasive species are one of the major threats to the maintenance of biodiversity, causing drastic changes in biological communities and affecting access to resources and the economy [1]. This problem also occurs in the Iberian Peninsula, with *Hakea decurrens* ssp. *physocarpa* WR Barker (Proteaceae) (Figure 1) being one of the species that are currently expanding. It was initially mistakenly identified in the Iberian Peninsula as *Hakea sericea* Schrad.& J.C. Wendl [2,3,4]. It belongs to the genus *Hakea*, which contains 154 species [2].

This species is an Australian shrub, specifically from dry and sclerophyllous forests in coastal regions of Southern Queensland and in the southwest of New South Wales, Australia. It is characterized by spiny, needle-like leaves, with small white flowers arranged in umbellate inflorescences and apiculate, ovoid, woody fruits that protect a winged seed [5]. It is a pyrophyte but non-resprouter shrub with high resistance to drought and a high thermal amplitude [6,7], presenting evolutionary strategies that encourage its spread and survival: its fruits are serotinous (they accumulate mature and viable seeds until the external conditions, fire, or the death of the plant itself trigger their spread by the wind) and resistant to heat and herbivory [8]. Another evolutionary characteristic is that this plant has proteoid roots (groupings of pilous roots along the main roots, increasing the contact surface up to 300-fold), which favors the capture of nutritional and water resources in poor and dry soils, thereby altering the edaphic conditions for the rest of the species [9].

*H. decurrens* subsp. *physocarpa* is considered a very dangerous invasive species worldwide [4], forming extensive and dense monospecific stands that exclude native plant species and change the composition of the community, including the associated fauna [10].

Invasive species, such as *H. decurrens* subsp. *physocarpa*, have morphological, physiological, and ecological features that facilitate their colonization and establishment outside of their natural habitats. One of these characteristics is allelopathy, i.e., an interaction that grants these species a clear competitive advantage [11]. Allelopathy is an interaction mediated by a plant’s production of chemical compounds that negatively affects the growth and development of other plants [12]. Allelochemicals produced by invasive species have a negative effect on the growth and/or reproduction of native species [13]. Different studies have associated the invasive capacity of a species with its allelopathic capacity [14]. For instance, Kalisz et al. [15] analyzed the allelopathic capacity of 524 invasive species, and the results showed that 51.4% of these species were allelopathic. Furthermore, this characteristic was widely distributed among the different families of invasive species.

The invasion of species mediated by allelopathy is supported by the novel weapons hypothesis (NWH), formulated by Callaway and Ridenour [16]. This hypothesis states that the compounds released by invasive species are new to autochthonous species; thus, native species are very sensitive to compounds from non-native species [17,18,19]. Different studies have shown that native species suffer more negative effects from invasive species (due to the concentration or proportion of allelochemicals) than from native species [20,21].

To date, no studies have attributed allelopathic activity to *H. decurrens* subsp. *physocarpa*, and the compounds derived from secondary metabolism that could play this role are unknown. In addition to the previously described characteristics that justify the invasive capacity of *H. decurrens* subsp. *physocarpa*, it is especially important to know the allelopathic potential of *H. decurrens* subsp. *physocarpa* and the compounds that would be involved in this interaction, with the aim of determining the ecological relationships that occur in habitats invaded by this species and contributing to its eradication.

Furthermore, it is important to highlight that the allelochemicals produced by one species may vary quantitatively and qualitatively in individuals of the same population throughout the year [22,23]. This is because the synthesis of these compounds depends on different environmental conditions [24,25,26], such as light intensity, light quality, water stress, and temperature [27]; thus, the allelopathy of a species may depend on the conditions under which it is found.

Therefore, the aim of the present study was to quantify the allelopathic activity of the aqueous extracts of *H. decurrens* subsp. *physocarpa* leaves and identify and quantify the compounds that are present in these aqueous extracts. The leaves were collected in different seasons to evaluate changes in the phytotoxic potential of the species.

## 2. Results

### 2.1. Bioactivity Test on Lactuca sativa

**Figure 2:**
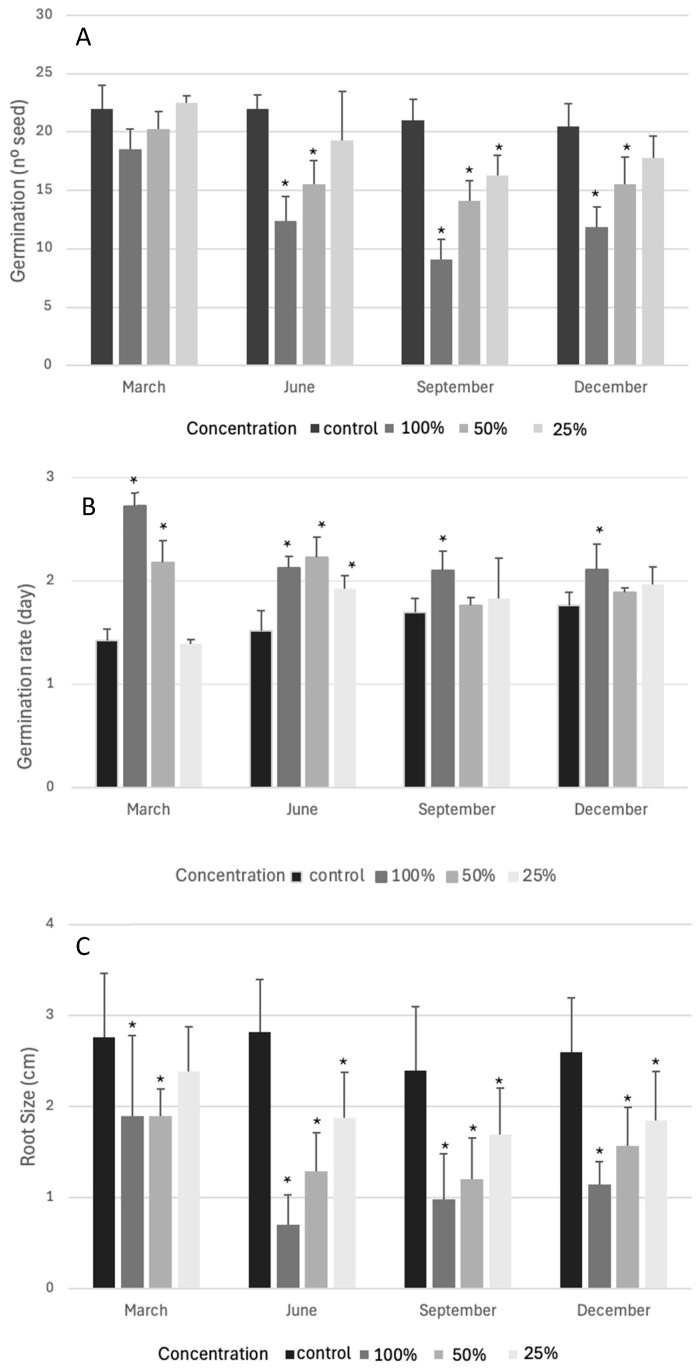
Effects of aqueous extracts from *H. decurrens* subsp. *physocarpa* leaves collected in March, June, September, and December on germination (**A**), germination rate (**B**), and root size (**C**) of *L. sativa* at 100%, 50%, and 25% concentration. *: Significant difference compared to control (Mann–Whitney test, *p* < 0.05). N = 4.

The effects of the aqueous extract from the leaves of *H. decurrens* subsp. *physocarpa* on germination, germination rate, and root size in *L. sativa* are shown in Figure 2. Germination (Figure 2A) was significantly inhibited (*p* < 0.05 M-W test) by the three concentrations of the aqueous extracts of the samples gathered in September; by contrast, the samples collected in March did not significantly inhibit germination at any of the three concentrations analyzed. The aqueous extracts derived from the leaves gathered in June and December exerted significant inhibition when analyzed at 100% and 50% concentration, but not at 25% concentration.

The germination rate (average number of days required for germination, Figure 2B) was significantly increased (*p* < 0.05 M-W test) by the extracts of all four samples at the maximum concentration. This significant negative effect was maintained, even at the lowest concentration, for the samples gathered in June and for the extract of the samples collected in March at 50% concentration.

Among the three parameters, root size was the most negatively affected (Figure 2C), presenting a significantly negative effect (*p* < 0.05 M-W test) for all samples and at all three concentrations analyzed, except for the lowest concentration of the aqueous extract from leaves that were collected in March.

To obtain more precise information about the effect of the aqueous extracts of the different samples, we calculated the I_50_ value, which is the concentration required for a 50% inhibition of the germination and root size of the test plants in the assay. I_50_ was determined by regression analysis, and as can be observed in Table 1, the concentration required to inhibit 50% of the germination ranged from 0.33 g/mL of the extract from samples gathered in March to 0.08 g/mL of the extract from samples collected in September. Root size was negatively affected by 50% by a range of 0.13 g/mL of the samples collected in March and by 0.05 g/mL of the samples collected in June.

### 2.2. HPLC Phytochemical Analysis of the Aqueous Extract of H. decurrens subsp. physocarpa

**Figure 3:**
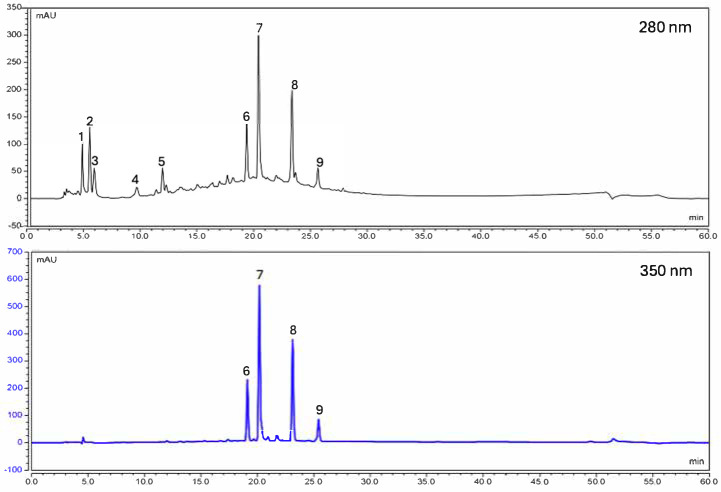
HPLC chromatogram of aqueous extract of *H. decurrens* subsp. *physocarpa* leaves at 280 and 350 nm. 1: arbutin; 2: mesaconic acid; 3: isotachioside; 4: 1-O-vanilloyl-beta-D-glucose; 5: syringic acid-4-beta-D-glucopyranoside; 6: quercetin 3-robinobioside-7-glucoside; 7: quercetin 3-rhamninoside; 8: rutin; 9: isorhamnetin-3-O-rutinoside.

Figure 3 shows a chromatogram of the compounds in the aqueous extract of the leaves of *H. decurrens* subsp. *physocarpa*, obtained by HPLC. The following phenolic compounds were identified: glycosylated hydroquinone (arbutin), carboxylic acid derived from ferulic acid (mesaconic acid), phenolic glycoside (isotachioside), vallinic acid (1-O-vanilloyl-beta-D-glucose), hydrolizable tannin (syringic acid-4-beta-D-glucopyranoside), and four flavonoids (quercetin 3-robinobioside-7-glucoside, quercetin 3-rhamninoside, rutin, and isorhamnetin-3-O-rutinoside).

As is described in Table 2, quercetin 3-rhamninoside and rutin represent the major compounds in the extract, at 29.3% and 20.2%, respectively, followed by quercetin 3-robinobioside-7-glucoside, mesaconic acid, and arbutin, at 11.9%, 9.1%, and 8.4%, respectively. The rest of the compounds were found at a lower percentage, ranging between 3.2% for 1-O-vanilloyl-beta-D-glucose and 6.7% for syringic acid-4-beta-D-glucopyranoside.

The amounts of each of these compounds, as well as their total sum, in each of the samples collected throughout the year are shown in Table 3. As can be observed, there is a quantitative variation among the samples: those gathered in December have the greatest representation, followed by those gathered in September, March, and June.

### 2.3. Correlation Between the Compounds Quantified in the Aqueous Extracts of H. decurrens subsp. physocarpa and Bioactivity Parameters: Germination, Germination Rate, and Root Size

With the aim of establishing whether the compounds present in the aqueous extract of *H. decurrens* subsp. *physocarpa* are involved in the phytotoxicity on *L. sativa*, we evaluated the correlation between the concentration of these compounds in the aqueous extracts quantified by HPLC and the quantified parameters: germination, germination rate, and root size. Table 4 shows the Pearson correlation coefficients and their degree of significance. As can be observed, there was a significant correlation between the effect on germination and the compounds quercetin 3-robinobioside-7-glucoside, quercetin 3-rhamninoside, isorhamnetin-3-O-rutinoside, arbutin, isotachioside, 1-O-vanilloyl-beta-D-glucose, and syringic acid-4-beta-D-glucopyranoside. Germination rate presented a significant correlation with arbutin, mesaconic acid, and 1-O-vanilloyl-beta-D-glucose. Lastly, root size was negatively and significantly correlated with arbutin and syringic acid-4-beta-D-glucopyranoside.

It is important to highlight that arbutin showed a significant correlation with the three parameters quantified, indicating the possible involvement of this compound in the global phytotoxic activity of this species.

## 3. Discussion

The results obtained in this work show a negative effect of the aqueous extracts of the leaves of *H. decurrens* subsp. *physocarpa* on the germination and growth of *L. sativa*. The extracts inhibited the quantified parameters with a concentration-dependent effect. This non-native species presented the same negative effect as other invasive species that are distributed in the same habitat, such as *Acer negundo*, *Salix babylonica*, and *Acacia dealbata* [28]. These species exert a negative effect on *L. sativa* with concentrations of aqueous extracts like those assayed with *H. decurrens* subsp. *physocarpa*, supporting the idea that allelopathy is one of the characteristics of invasive species that allow them to establish themselves outside of their natural habitat [15].

Among the quantified parameters, root size was the most affected, thereby corroborating other studies, which report that the most sensitive morphological parameter for the evaluation of the allelopathic activity of a species is root size [29,30]. Germination is a very important component for the quantification of allelopathy, although the effect on root size may be equally or even more relevant, since root development is essential for seedling survival, negatively affecting their competitive capacity [31]. It is also important to highlight the delay in the germination rate of *L. sativa*, which was observed when the seeds were treated with the aqueous extracts of *H. decurrens* subsp. *physocarpa*. Although the effect on this parameter was less negative than that on the other two parameters, a delay in germination affects the establishment of species in natural conditions, as they may waste the favorable conditions for their successful establishment. Moreover, germination rate provides a more realistic perspective of the activity of these extracts as possible allelopathic agents in the natural medium and their possible use as natural herbicides [32].

The negative effect on germination and root size occurred with the extracts from samples gathered in March, June, September, and December. It should be noted that rainfall in the sampling area is present throughout the year [33], which implies that the leaching of leaves and their possible allelopathic activity would be maintained throughout the year. In addition to this, allelochemicals from plants are released into the environment by exudation from the roots, leaching from the stems and leaves, or the decomposition of plant material [34,35], Therefore, the study of the involvement of other organs, such as the roots, in the allelopathy of *H. decurrens* subsp. *physocarpa* could be useful to reinforce the allelopathic activity of this species. On the other hand, there were differences in the effect caused by each of them. Considering the values of I_50_ (Table 1), the extract obtained from the leaves gathered in September had a 4.2, 1.3, and 1.4 times more negative effect on germination than the extract obtained from the leaves collected in March, June, and December, respectively, and the extract of leaves gathered in June had a 2.3, 1.3, and 1.4 times more negative effect on root size than the extract of leaves collected in March, September, and December, respectively. These results reveal that the phytotoxicity of the leaves of *H. decurrens* subsp. *physocarpa* may depend on the time of the year. This seasonal variation in allelopathic activity has been demonstrated in other species, such as *Brachiaria brizantha* and *Pinus densiflora* [36,37]. It should also be noted that the concentrations of the aqueous extracts to obtain 50% inhibition of germination (0.08 to 0.33 g/mL) are similar to those obtained in trials with other invasive allelopathic species, such as *Rhus typhina* and *Impatiens glandulifera* [38].

In regard to the phytochemical composition, nine different compounds were quantified in the aqueous extracts through HPLC: arbutin, mesaconic acid, isotachioside, 1-O-vanilloyl-beta-D-glucose, syringic acid-4-beta-D-glucopyranoside, quercetin 3-robinobioside-7-glucoside, quercetin 3-rhamninoside, rutin, and isorhamnetin-3-O-rutinoside. Numerous studies have reported the involvement of phenolic compounds in the phytotoxicity of different species [39]. Compounds such as coumaric acid and its derivatives inhibit enzymes such as glucose phosphate isomerase, 6-phosphate dehydrogenase, and aldolase in the oxidative pentose phosphate pathway, causing a detrimental effect on plant growth [40]. Furthermore, p-coumarin is known for its inhibitory effect on seed germination and plant growth, and it also causes a deleterious effect on root growth by changing its morphological and physiological structure [41,42,43]. Similarly, it has been demonstrated that flavonoids such as quercetin can affect the electron transport system, causing an inhibition of substrate oxidation, thus disrupting the uptake of phosphate [44] and altering membrane permeability. Other phenolic compounds have been found to have a deleterious effect on nucleic acids and other cellular components such as ribosomes and the mitochondria [45,46].

It is important to determine the compound/s that cause the phytotoxicity attributed to the aqueous extracts of the leaves of *H. decurrens* subsp. *physocarpa*. The results of the correlation between the amounts of these compounds in the extracts and their effect on germination, germination rate, and root size (Tabla 4) may provide such information. From these results, it is worth highlighting that the two flavonol glycosides (quercetin 3-robinobioside-7-glucoside and quercetin 3-rhamninoside) showed a significant negative relationship with germination, as is the case for arbutin, isotachioside, 1-O-vanilloyl-beta-D-glucose, and syringic acid-4-beta-D-glucopyranoside. Of the latter, arbutin and 1-O-vanilloyl-beta-D-glucose also presented a significant relationship with germination rate. Once again, arbutin and syringic acid-4-beta-D-glucopyranoside showed a significant correlation with the negative effect on root size.

Among the flavonoids identified, only two of them showed a significant correlation with germination. In [47], it was demonstrated that flavonoids such as myricetin, luteolin, rutin, and (+)- catechin, which are present in the extract of leaves of *Juglans regia*, contributed significantly to its phytotoxicity on the germination of *Amaranthus retroflexus* L. and *Chenopodium álbum*, with catechin and luteolin being the two main allelochemicals responsible for inducing oxidative stress in the tested weeds.

The other compounds that could be involved in the phytotoxicity of *H. decurrens* subsp. *physocarpa* are arbutin, O-vanilloyl-beta-D-glucose, and syringic acid-4-beta-D-glucopyranoside. It is important to point out that O-vanilloyl-beta-D-glucose and syringic acid-4-beta-D-glucopyranoside were negatively correlated with two of the quantified parameters, and arbutin was negatively correlated with all three parameters.

Arbutin is a glycosylated hydroquinone that has been detected in approximately 50 families, with the families Asteraceae, Ericaceae, Proteaceae, and Rosaceae being the main sources of this compound. In another species of the genus *Hakea* (*Hakea saligna* L.), an important amount of the hydroquinone arbutin has been identified and quantified [48]. The interest in research on this hydroquinone lies mainly in its therapeutic properties as an antioxidant, anti-inflammatory, antimicrobial, and anti-cancer agent [48,49,50,51,52,53,54,55], as well as its involvement in phytotoxic activity on plant germination and growth. Arbutin has already been identified as a possible allelochemical acting on soil microorganisms [56] and has been proposed as one of the allelochemicals responsible for the allelopathic activity of *Arbutus unedo* and *Myrtus communis* [57].

Furthermore, the prevalence of this compound in soils has also been studied, with the aim of determining the real potential of arbutin as an allelopathic agent [58]. It has been demonstrated that, in non-sterilized soils, arbutin is transformed into hydroquinone and then into benzoquinone. Benzoquinone is more toxic than hydroquinone in comparative bioassays [59], and it persists in the soil for a long time, which supports the idea that arbutin could be an allelopathic agent.

Although there was a significant correlation between certain compounds present in the leaves of *H. decurrens* subsp. *physocarpa* and the quantified parameters, it would not be correct to attribute the allelopathic activity of this species to a specific compound. Different studies, such as one conducted for the quantification of the allelopathic activity of *A. unedo* and *M. communuis* [57,60], have reported that “the whole is greater than the sum of its parts”. The authors propose that arbutin is the main component responsible for the allelopathic activity of these species, although there are co-occurring compounds that may contribute to modulating and increasing its activity. In the same vein, the authors of [61] show that the effects of individual phenolic acids such as benzoic acid, p-hydroxybenzaldehyde, trans-cinnamic acid, p-hydroxybenzoic acid, vanillic acid, 3,4-dihydroxybenzoic acid, p-coumaric acid, and ferulic acid are insufficient to suppress the growth and germination of the assayed species, although their mixtures had a significant inhibitory effect. Another study that reported the same behavior is [62]. Its authors quantified the effect of an aqueous extract of *Delonix regia* on the growth of lettuce (*L. sativa*) and Chinese cabbage (*Brassica chinensis*). The compounds identified in the extract were chlorogenic acid, protocatechuic acid, gallic acid, 3,4-dihydroxybenzaldehyde, p-hydroxybenzoic acid, caffeic acid, and 3,5-dinitrobenzoic acid, and the allelopathic activity quantified depended on the combination of these compounds. This could be due to the fact that each compound has different mechanisms of action or different effects at the metabolic level; thus, the mixtures are much more active and trigger different and more drastic responses. Specifically, in our study, root size was the parameter that was most negatively affected by the aqueous extracts of *H. decurrens* subsp. *physocarpa*, although it showed the weakest correlation with the quantified compounds in the extracts. This could be because root size is very sensitive to these compounds, which, in addition to the combined action of all of them, leads to a much more drastic effect than that which could be derived from the amount of a particular compound.

## 4. Materials and Methods

### 4.1. Gathering of Materials and Sample Treatment

The collection of plant material was carried out in invaded areas of Extremadura (Spain) in the town of Valverde del Fresno (40°13′26″ N 6°52′47″ W). The average annual rainfall ranges between 1000 and 1200 mm/year and the average annual temperature is between 14 and 15 °C. The main vegetation accompanying *H. decurrens* subsp. *physocarpa* is *Pinus pinaster* with an understory of mostly *Cistus ladanifer*, *Arbutus unedo*, *Lavandula stoechas*, and *Calluna vulgaris* [33]. Throughout the year, four samples of leaves were collected in the months of March, June, September, and December. Samples were collected from different individuals with a size greater than 2 m, which were randomly selected. Leaves were taken from three different sites (vouchers were deposited in the Herbarium of the Instituto de Investigaciones Agrarias Finca La Orden—Valdesequera, CICYTEX-Junta de Extremadura (HSS 87165/87181/87203)) and then mixed to obtain approximately 1 kg of leaves. The samples were taken to the laboratory on the same day as the sampling was conducted, and they were left to dry at room temperature. When the leaves were dry enough, they were ground into a powder using an electric grinder and were then kept in the dark at room temperature.

### 4.2. Preparation of the Aqueous Extracts

Aqueous solutions were prepared for bioassays. Dry leaves were mixed with distilled water (1:10 *w*/*v*) [38,63] and were left stirring at room temperature for 24 h. Then, the samples were filtered, and 3 concentrations were prepared. The original solution (100%) was diluted with distilled water to obtain concentrations of 50% and 25%.

### 4.3. Bioassays

#### 4.3.1. Bioassays on *Lactuca sativa* Germination

The allelopathic potential of *H. decurrens* subsp. *physocarpa* was quantified using *L. sativa* as the target species. This species is considered a comparable indicator of allelopathy among species due to its rapid germination [64] and due to being commonly used in phytotoxic studies [65,66].

A total of 25 seeds were placed in Petri dishes with filter paper (4 replicates for each concentration and control). Then, 5 mL of the dilution was added to each dish, and the dishes were sealed with Parafilm. For the control, distilled water was added.

The dishes were randomly placed in a culture chamber at 20/15 °C with a photoperiod of 14 h of light and 10 h of darkness for three days. Germination was quantified daily in each dish.

With these data, the following parameters were obtained:

- Germination: Number of germinated seeds.
- Germination rate: The germination rate is an arithmetic mean that indicates the days required for germination [67]. It was calculated using the formula cited by [68], as follows:

*GR* = *N*_1_*G*_1_ + *Nn*_2_*G*_2_ + …… + *NnGnG*_1_ + *G*_2_ + …… + *Gn* = ∑*ni* = 1*NiGi*∑*ni* = 1*Gi*

where *GR* is the germination rate; *N_1_*, *N_2_*, …, *Nn* represent the number of days from the beginning of the germination test; and *G_1_*, *G_2_*, …, *Gn* represent the number of germinated seeds on day n.

#### 4.3.2. Bioassays on *L. sativa* Root Size

For this bioassay, germinated seeds were used. To this end, lettuce seeds were placed in Petri dishes with distilled water in the culture chamber. Immediately after germination, a total of 25 germinated seeds were placed in Petri dishes with filter paper (4 replicates for each concentration and control). Then, 5 mL of the dilution was added to each dish, distilled water was added to the control, and the dishes were sealed with Parafilm. The dishes were randomly placed in the culture chamber at 20/15 °C with a photoperiod of 14 h of light and 10 h of darkness. After 10 days, the seedlings were extracted, and the length of their roots was recorded.

### 4.4. Identification and Quantification of Phenolic Compounds

#### 4.4.1. Identification: UHPLC/Q-TOF MS Method

Analysis of extracts was carried out in a UHPLC apparatus (Agilent 1260, Agilent Technologies, Santa Clara, CA, USA) with DAD (Agilent G7117A) and an Accurate Mass QTOF mass analyzer (Agilent 6520) with an atmospheric pressure electrospray ionization (ESI). It features a quadrupole time-of-flight (QTOF) mass analyzer that delivers high mass resolution and mass accuracy. Separation was performed in a Spherisorb C18 (150 × 4.6 mm) reversed-phase column at a rate of 0.5 mL/min. The mobile phase consisted of 0.1% formic acid in water (A) and 0.1% formic acid in acetonitrile (B) using a gradient as follows: initially, 95% A; 10 min 85% A; 20 min 80% A; 40 min 60% A; 50 min 20% A; 55 min 10% A; 65 min 95% A. Data were acquired using a negative ion mode with a mass range of 100 to 1700 *m*/*z* and using a source temperature of 300 °C and a gas flow of 10 L/h.

The molecular formulas proposed by the MassHunter Workstation software version 4.0 for the different signals obtained in the MS experiments were compared with previously reported phenolic compounds, and a maximum error of 10 ppm was accepted. Mass measurement error (mass accuracy) was calculated according to Brenton and Godfrey [69]: Difference between an individual measurement and the true value ∆Mi (in ppm, parts per million) = (M measured − M calculated) × 10^6^/M calculated, where M measured is the measured mass in QTOF-MS and M calculated is the exact calculated mass according to the molecular formula of the compound.

#### 4.4.2. Quantification: HPLC-DAD Method

The quantifications were performed with an HPLC apparatus (Agilent 1260, Agilent Technologies, Santa Clara, CA, USA) with DAD (Agilent G7117A). A total of 20 mL filtered extract of each sample was injected into a Spherisorb C18 (150 × 4.6 mm) reversed-phase column at a rate of 0.5 mL/min. The mobile phase consisted of 0.1% formic acid in water (A) and 0.1% formic acid in acetonitrile (B) using a gradient as follows: initially, 95% A; 10 min 85% A; 20 min 80% A; 40 min 60% A; 50 min 20% A; 55 min 10% A; 65 min 95% A.

Chromatograms were recorded at a wavelength of 350 nm and 280 nm. Concentrations of the compounds (N = 3) were estimated from a standard curve (0.001; 0.005; 0.05; 0.01; and 0.1 mg/mL) using gallic acid or quercetin 3-O-rutinoside (rutin). The results are expressed in mg of equivalents per g of dry weight.

### 4.5. Statistical Analysis

To assess the effect of the treatment on the different variables, the Mann–Whitney non-parametric test was used accordingly. The correlation coefficients were calculated with Pearson’s test. All statistical analyses were carried out using the SPSS statistical software (29.0.1.0). Statistical significance was established at *p* < 0.05.

## 5. Conclusions

In conclusion, this study shows for the first time that the leaves of the invasive species *H. decurrens* subsp. *physocarpa* present allelopathic potential. This phytotoxic activity has been shown to be present throughout the year. Therefore, allelopathy should be considered to be one of the mechanisms that allow for the invasiveness of this species. In addition, the phytochemical composition of these extracts has been analyzed, reporting the presence of a group of phenolic compounds and their derivatives. The correlation found between the amount of these compounds and the phytotoxic activity could suggest that these compounds are involved in the phytotoxicity of this species. Future studies with pure compounds would be necessary to know the degree of involvement of each of them.

## Figures and Tables

**Table 1:** I_50_ value (concentration causing 50% of growth inhibition) of aqueous extract of *Hackea decurrens* subsp. *physocarpa* leaves (g/mL) for germination and root size of *L. sativa*.

		I_50_ (g/mL)
	Germination	Root Size
March	0.33	0.13
June	0.1	0.05
September	0.08	0.07
December	0.1	0.08

**Table 2:** Compounds identified in the aqueous extract of the leaves of *H. decurrens* subsp. *physocarpa* and the percentage contribution of each of them. Data recorded using HPLC-DAD and UHPLC coupled to quadrupole time. The values of the percentages of the areas are the mean of the 4 samples collected throughout the year (4 samples for three replicates; N = 12).

Compounds	Molecular Formula	Rt HPLC-DAD (min)	Measured [M-H]-	Exact Mass (calc.)	Ppm Value	Peak Area %
Arbutin	C12H16O7	4.5	271.0821	271.0823	0.73	8.4
Mesaconic acid	C5H6O4	5.2	129.2000	129.1930	−5.14	9.1
Isotachioside	C13H18O8	5.6	301.0936	301.0929	−2.35	5.7
1-O-vanilloyl-beta-D-glucose	C14H18O9	9.4	329.0883	329.0878	−1.5	3.2
Syringic acid-4-beta-D-glucopyranoside	C15H20O10	11.7	359.0984	359.0984	−0.8	6.7
Quercetin 3-robinobioside-7-glucoside	C33H40O21	19.1	771.2015	771.1989	−3.33	11.9
Quercetin 3-rhamninoside	C33H40O20	20.2	755.2049	755.204	−1.17	29.3
Rutin	C27H30O26	23.1	609.1467	609.1461	−0.97	20.2
Isorhamnetin-3-O-rutinoside	C28H32O16	25.4	623.1626	623.1618	−1.35	5.4

Rt (min), retention times obtained from HPLC-DAD and UHPLC-DAD; [M-H]-, base ion at negative mode. Percentage peak area from HPLC-DAD chromatograms was acquired at 280 nm.

**Table 3:** The amounts of each of the compounds (mg of equivalents (gallic acid or rutin) per g of dry weight) present in the aqueous extract derived from the leaves of *H. decurrens* subsp. *physocarpa*. Values are the means of three replicates ± standard deviation.

Compounds (mg/gDW)	March	June	September	December
Arbutin	0.27 ± 0.01	0.22 ± 0.015	0.27 ± 0.001	0.25 ± 0.016
Mesaconic acid	0.29 ± 0.009	0.23 ± 0.012	0.23 ± 0.009	0.35 ± 0.011
Isotachioside	0.16 ± 0.0002	0.14 ± 0.003	0.17 ± 0.005	0.23 ± 0.009
1-O-vanilloyl-beta-D-glucose	0.10 ± 0.001	0.08 ± 0.0003	0.11 ± 0.004	0.09 ± 0.0002
Syringic acid-4-beta-D-glucopyranoside	0.18 ± 0.009	0.19 ± 0.0001	0.28 ± 0.008	0.17 ± 0.006
Quercetin 3-robinobioside-7-glucoside	0.37 ± 0.013	0.21 ± 0.0003	0.67 ± 0.01	0.90 ± 0.015
Quercetin 3-rhamninoside	1.05 ± 0.01	0.49 ± 0.002	1.62 ± 0.003	2.14 ± 0.016
Rutin	0.90 ± 0.009	0.51 ± 0.0001	0.74 ± 0.017	1.50 ± 0.005
Isorhamnetin-3-O-rutinoside	0.22 ± 0.002	0.15 ± 0.0006	0.24 ± 0.01	0.37 ± 0.014
Total compounds	3.54	2.20	4.32	5.99

**Table 4:** Pearson’s correlation coefficient and significance (*p* value) between the amount of each of the compounds in the aqueous extracts of the leaves of *H. decurrens* subsp. *physocarpa* and the values of the quantified parameters (germination, germination rate, and root size). (* *p* < 0.05; ** *p* < 0.005).

	Germination		Germination Rate		Root Size	
	Pearson Correlation	Sig	Pearson Correlation	Sig	Pearson Correlation	Sig
Quercetin 3-robinobioside-7-glucoside	−0.644 *	0.024	0.245	0.443	−0.370	0.236
Quercetin 3-rhamninoside	−0.613 *	0.034	0.291	0.358	−0.335	0.287
Rutin	−0.445	0.147	0.440	0.152	−0.321	0.309
Isorhamnetin-3-O-rutinoside	−0.583 *	0.047	0.443	0.149	−0.427	0.166
Arbutin	−0.642 *	0.024	0.635 *	0.027	−0.578 *	0.049
Mesaconic acid	−0.522	0.081	0.601 *	0.039	−0.493	0.103
Isotachioside	−0.645 *	0.024	0.446	0.146	−0.523	0.081
1-O-vanilloyl-beta-D-glucose	−0.612 *	0.035	0.597 *	0.041	−0.530	0.076
Syringic acid-4-beta-D-glucopyranoside	−0.795 **	0.002	0.485	0.110	−0.689 *	0.013

## Data Availability

No new data were created or analyzed in this study. Data sharing is not applicable to this article.
